# How does workplace support promote postdoctoral career growth? A conservation of resources perspective

**DOI:** 10.3389/fpsyg.2024.1294982

**Published:** 2024-01-25

**Authors:** Xueyan Li, Anqi Hu, Hongfeng Song, Zhimei Wang

**Affiliations:** ^1^School of Economics and Management, Beijing Forestry University, Beijing, China; ^2^Business School, Central University of Finance and Economics, Beijing, China

**Keywords:** postdoctoral researcher, workplace support, psychological capital, work-life balance, career growth, conservation of resources theory

## Abstract

**Background:**

Postdoctoral researchers are critical to scholarly advancements, and promoting postdoctoral career growth is an endogenous path to help postdocs break through the “encircled city of scientific research”. However, further research is needed to fully explore the mechanisms that connect workplace support to postdoctoral career growth.

**Methods:**

Drawing from the Conservation of Resources theory, this study proposes a chain mediation model that demonstrates how workplace support enhances career growth by connecting psychological capital with work-life balance. To understand the motivation and career growth of postdocs in China, we conducted two questionnaires in 2021 and 2023 with the support of relevant stations.

**Results:**

Analyzing 367 questionnaires from Chinese postdocs, our research indicates that workplace support has a positive impact on career growth. Additionally, both psychological capital and work-life balance are key factors that contribute to career growth, serving as separate mediators and as part of a chain of mediators.

**Discussion:**

This study validates the appropriateness of the Conservation of Resources theory in the study of the influence mechanism of postdoctoral career growth and proposes targeted strategies for academic institutions to improve support systems, promoting more effective career development pathways.

## 1 Introduction

In an era where innovation is the driving force behind global advancements, postdoctoral researchers stand at the forefront, shaping the future of science and technology. Postdocs possess robust potential for national scientific innovation and are essential to talent-driven national strengthening and innovation-led development strategies (Liu and Yuan, 2016). In this context, it is of great theoretical value and practical significance to answer the question of “what factors can contribute to the career growth of the postdoctoral units and the specific path to enhancement” through empirical research.

Postdoctoral researchers’ career growth is multidimensional, highlighting enhancements in professional capability and deepening academic career identities. Postdocs face multiple pressures in becoming independent researchers: the conflict between instrumental and value rationality, the confrontation between the roles of learner and researcher, and the tension between academics and market rules (Zhang, 2022). Drawing from the Conservation of Resources (COR) theory, postdocs who struggle to navigate the complexities of academic socialization may experience a feeling of resource erosion. This can result in higher levels of stress and a downward spiral of resource losses, ultimately hindering their career growth (Hobfoll, 1989).

Numerous studies have confirmed the critical importance of support from the workplace in facilitating the postdoctoral career growth (CG) (Lovitts, 2002; Gardner, 2008). Workplace support (WS) is recognized as an important influencing factor, but its mechanism of action has yet to be fully explained and empirically tested. Recent studies highlight the importance of fostering positive psychological states and reconciling work and personal life demands. These findings suggest that psychological capital (PsyCap) and work-life balance (WLB) could be critical variables in assisting postdocs to achieve better career growth (Abendroth and Den Dulk, 2011; Caesens et al., 2020; Saleem et al., 2022a). Addressing this knowledge gap, we propose that WS enhances CG by increasing personal resources (notably PsyCap) and improving well-being (particularly regarding WLB).

By conducting a questionnaire survey in 2023 with the support of the postdoctoral station, we attempt to answer the following questions: first, what is the correlation between WS and postdoctoral CG? Second, through what pathways and mechanisms does WS influence postdoctoral CG? This study offers three main contributions. First, supportive work environments in Chinese postdoctoral units play a crucial role in the career growth of postdocs, mentors, institutions, and peers can provide resources that support postdocs’ mental well-being and help them advance professionally and academically. Second, building on previous studies, we emphasize the central role of PsyCap and WLB as mechanisms connecting WS and CG, thus clarifying the positive impact of WS and advancing our understanding of its underlying processes. Third, our study provides insights for postdoctoral institutions, highlighting the potential benefits of offering work resources, ranging from mentorship guidance and colleague identification to institutional support.

## 2 Theory and hypotheses

The COR theory suggests that individuals strive to acquire, retain, foster, and increase resources perceived as valuable. Stress is intricately linked to specific contexts (Hobfoll et al., 1990), and resources are central to the genesis of stress and the strategies for coping with it. Postdoctoral challenges in their professional and personal lives can be conceptualized as potential resource losses and the continual effort to acquire new resources. Individuals deficient in such resources will find it difficult to manage stress effectively, thus inhibiting their optimal career growth (Hobfoll, 1989).

The COR theory clarifies the origin of stress and coping mechanisms primarily through the constructs of the loss spiral and the gain spiral, gradually establishing itself as a central theory in organizational behavior studies, encompassing workplace psychology and related behaviors (Liao et al., 2022). Specifically, the loss spiral implies that when an individual is under stress and cannot effectively stop resource depletion or secure timely compensation, the erosion of resources intensifies (Hobfoll, 1989). For instance, postdocs may compromise their health due to academic pressures, affecting their work efficiency and family harmony. On the contrary, the gain spiral suggests that when individuals have large amounts of resources, they have more significant opportunities to secure new resources by investing in existing ones, leading to the accumulation of resources and further growth, similar to a “compound interest” effect (Hobfoll, 2001). A case in point: a successful postdoctoral publication could lead to a more confident work attitude and broader research opportunities.

The postdoctoral phase represents a crucial time for young scholars to accumulate resources and seek future developmental opportunities. Several stress-related factors are present throughout this process: academic pressures from mentors, research teams, and institutions (with postdocs expected to produce high-quality publications, secure research funding, and complete projects within limited timeframes) (Hangel and Schmidt-Pfister, 2017; Haven et al., 2019). Career development pressures stemming from the job market, industry competition, and financial demands (with postdocs needing to prepare for their careers in a constrained timeframe) (Sauermann and Roach, 2016; Roach and Sauermann, 2017). Moreover, pressures from work-life conflicts and health issues (requiring postdocs to juggle multiple roles) (Martinez et al., 2007; Mason et al., 2009). These factors expedite resource depletion and are highly susceptible to inducing a pessimistic mental state and work-life imbalance, aligning closely with the foundational tenets of COR theory. Based on the classification by Ten Brummelhuis and Bakker (2012) within the COR framework, mentor, institutional, and peer support (collectively called WS) can be viewed as external resources. Meanwhile, PsyCap and WLB are intrinsic individual resources. Although internal and external resources are appropriately identified, the interactions between internal and external resources and the results of those interactions need to be explored in depth, which is the research gap that needs to be addressed in this paper. Thus, grounded in the COR theory, we explore the interrelations between WS, PsyCap, WLB, and CG.

### 2.1 Workplace support and postdoctoral career growth

Social support originated in community psychology, referring to an individual’s experiences of respect, care, and assistance within their social network (Taylor et al., 2004). Postdoctoral work requires specialized expertise, ample resources, and collaborative teamwork, making support from mentors, institutions, and peers essential. Mentor support refers to supportive behaviors mentors exhibit throughout the academic and life journey (Crisp and Cruz, 2009). Institutional support refers to supportive behaviors where the institution values the contributions of its fellows, offering essential resources to navigate life and work challenges (Eisenberger et al., 1986; Thompson et al., 2020). Peer support is functional assistance researchers receive through their social networks (Cohen and Willis, 1985; Beehr et al., 2000), specifically from peers of similar rank and status within formal organizations (Jian and He, 2022). They are collectively termed as “WS” in our study. As an academic career, the postdoctoral CG includes growth in employability and skills and involves academic progress and self-fulfillment. This paper delineated CG regarding professional capability development and academic career identity: postdocs continually reinforce and expand their professional skills and knowledge throughout their academic career, deepening their identification and value perception of their research.

Postdocs encounter a range of multidimensional challenges. Based on the COR theory, postdocs may face psychological stress without timely resource replenishment, hindering their career growth (Hobfoll, 2001). The importance of WS for new career development has been demonstrated in many different career situations. For newly recruited nurses and doctors (Smythe and Carter, 2022; Ranse et al., 2023), secondary school teachers (Foster-Collins et al., 2023), and social workers (Tang and Li, 2021), empirical studies are showing that WS is effective in helping them to improve their role clarity and job competence, reduce role pressure and job burnout, and promote adaptation and growth.

For young postdoctoral scholars, this stage is a process of human capital accumulation and signal capital investment (Zhang and Liu, 2019). This stage marks the beginning of postdoctoral academic careers, and WS is crucial. The most critical sources of WS for postdocs are mentors, institutions, and peers. Existing studies on mentor, institutional, and peer support roles have also yielded abundant findings. Past research has illustrated that mentor support can significantly influence postdoctoral subjective well-being and work enthusiasm (Gloria and Steinhardt, 2017), enhance postdoctoral research competence and outcomes, help them fulfill role expectations (Jaeger and Dinin, 2018), and further boost their employability and career growth (Nowell et al., 2020). An elevated level of institutional support typically produces positive attitudes and behaviors from employees. In an environment where postdocs feel respected, trusted, and supported, they are better equipped to use internal and external resources to manage crises and handle stress (Rhoades and Eisenberger, 2002; Riggle et al., 2009; Kurtessis et al., 2017). Peer support benefits employee well-being, improving job satisfaction and mental health (Ducharme and Martin, 2000). Establishing supportive relationships with peers facilitates the exchange of information and resources, leading to higher organizational commitment (Sias, 2008). Therefore, based on the theory of social support, this study hypothesizes that the perceived WS by postdoctoral researchers will directly and positively influence their level of CG:

*Hypothesis 1 (H1)*. WS is positively associated with CG of postdoc.

### 2.2 Mediating role of psychological capital

Luthans (2002) posited that PsyCap represents a positive psychological force that can stimulate affirmative attitudes and behaviors, driving personal and professional growth and enhancing competitive advantage. It is compartmentalized into four dimensions: Optimism, Resilience, Self-efficacy, and Hope (Luthans et al., 2007). Combined with COR theory, individuals with abundant resources seek to acquire new resources (Hobfoll, 2002). Postdocs can receive more resource support from their mentors, institutions, and peers and accumulate rich personal resources when they receive high workplace support. Individuals are more sensitive to resource acquisition (Halbesleben et al., 2014). They will strive to utilize their work resources to construct and accumulate internal psychological resources to maintain and promote their progress.

Supportive work environments can help alleviate the stress experienced by postdoctoral researchers (Bakker et al., 2014) and cultivate PsyCap (Caesens et al., 2020). The positive effects of supportive factors on PsyCap have been verified in relevant empirical studies. For example, Teacher support and a family environment can reduce academic procrastination by improving students’ PsyCap (Zeng et al., 2022). The sense of organizational support is positively associated with PsyCap of college students (Wang et al., 2023). Perceived peer support, an essential contextual resource, has also been shown to help diminish an individual’s workload and enhance work well-being (Peng et al., 2021). Individuals with high levels of PsyCap are motivated and nurtured by intrinsic motivation to become more driven and fulfilled and actively pursue their academic and professional goals. PsyCap has been shown to have a positive correlation between the outcome variables of supervisor-rated job performance (Abbas et al., 2014), academic performance (Carmona-Halty et al., 2021), and work engagement (Saleem et al., 2022b).

Social cognitive theory suggests that environmental factors can only act on behavior through the internal aspects of the individual (Bandura, 1977). Based on the above analysis, PsyCap (internal aspects) may play a bridge transduction role between WS (environmental factors) and CG (behavior). We proposed the following hypotheses:

*Hypothesis 2a (H2a)*. WS is positively associated with PsyCap of postdoc.

*Hypothesis 2b (H2b)*. PsyCap is positively associated with CG of postdoc.

*Hypothesis 2c (H2c)*. PsyCap mediates the impact of WS on CG.

### 2.3 Mediating role of work-life balance

A widely accepted definition by Heery and Noon (2001) suggests that WLB is achieved when the cohort engaged in paid employment can exercise a degree of control over their work time, place, and manner, minimizing conflict and establishing equilibrium between work and non-work domains. This balance is considered a collective societal interest and a fundamental human right. An imbalance, or conflict, implies that work duties hinder their ability to fulfill life roles or vice versa (Greenhaus and Beutell, 1985; Frone, 2000; Amstad et al., 2011). Postdoctoral fellows experience conflicts between their professional and personal responsibilities and achieving work-life balance is crucial for them (Pitt et al., 2021).

Some relevant studies have shown that perceived support promotes WLB in individuals. Research has shown that social support from the workplace and family can improve employees’ WLB and psychological well-being (Russo et al., 2016). Perceived organizational support significantly predicts workers’ WLB (Amazue and Onyishi, 2016), and this facilitation is positively moderated by organizational commitment (Sheikh, 2022). Mentor support and guidance in the academic research environment are considered critical for students to achieve WLB (Durbin et al., 2019). Emotional and instrumental support from coworkers and supervisors significantly impact an individual’s WLB (Bradley et al., 2023; Uddin et al., 2023). In many works of literature, the positive impact of WLB has been studied. WLB is found to be directly and positively correlated with work engagement (Wood et al., 2020), job satisfaction (Irawanto et al., 2021), job commitment (Aruldoss et al., 2021), and well-being (Pace and Sciotto, 2021). For postdocs, providing a supportive and healthy work environment benefits their well-being and fosters their academic and professional growth. Based on the above considerations, we posited the following hypotheses:

*Hypothesis 3a (H3a)*. WS is positively associated with WLB of postdoc.

*Hypothesis 3b (H3b)*. WLB is positively associated with CG of postdoc.

*Hypothesis 3c (H3c)*. WLB mediates the influence of WS on CG.

### 2.4 Chain mediating role of PsyCap and WLB

Postdoctoral researchers face multiple pressures to balance academic research, assessment standards, family responsibilities, and financial needs. Thus, WLB is often challenging due to various pressures. PsyCap is a positive mindset that helps individuals assess situations optimistically and increases the chances of success by promoting positive effort and resilience (Luthans et al., 2007). Recent studies have consistently shown a positive correlation between PsyCap and WLB attainment. Allameh et al. (2018), Wardani and Anwar (2019), and Vaziri et al. (2022) have found that individuals with higher PsyCap are more likely to achieve better WLB.

Based on COR theory (Hobfoll et al., 2018), Psycap, as a positive personality trait, serves as a crucial intrinsic resource. When individuals have a higher sense of self-efficacy, are hopeful about things, and possess optimism and good resilience, they can access resources to better deal with the demands and conflicts and achieve WLB. As a critical work resource, WS can activate the “gain spiral” effect, generating a resource “compounding” effect based on sufficient work resources, increasing an individual’s intrinsic psychological resources and improving WLB. This continuous growth of resources helps postdocs accumulate positive psychological energy, enhances their resilience, and facilitates their achievement of career growth. In previous studies, WLB has also been shown to be positively related to organizational commitment and job satisfaction outcome variables (Carlson et al., 2009; Mas-Machuca et al., 2016; Oyewobi et al., 2022). In academic career contexts, WLB is often seen as a positive buffer against the stresses of academic research (Bozeman and Gaughan, 2011). From those above, the following hypotheses were proposed:

*Hypothesis 4a (H4a)*. PsyCap is positively associated with WLB of postdoc.

*Hypothesis 4b (H4b)*. WS influences CG through a chain-mediating effect of PsyCap and WLB.

**Figure 1:**
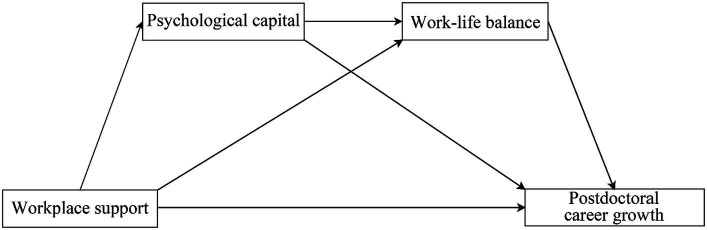
Mechanisms of workplace support (WS) in postdoctoral career growth (CG).

In summary, this study constructed a theoretical model of the influence mechanism of WS on postdoctoral CG, as shown in Figure 1.

## 3 Materials and methods

### 3.1 Data sources

To understand the motivation and career growth of postdocs in China, we conducted two questionnaires in 2021 and 2023 with the support of relevant stations. In 2021, we issued 4,000 questionnaires and received 3,473 valid responses (an 86.83% response rate) to assess the level of work environment support. In 2023, we advanced our previous research with a follow-up survey of 800 Chinese postdocs, receiving 769 responses (a 96.13% response rate), to further explore their psychosocial status, work-life situation, and career growth level. After two sample matches, 367 valid samples were obtained. The returned questionnaires underwent outlier detection using SPSS 27.0. From a gender perspective, 247 respondents were male, representing 67.30%, while 120 were female, representing 32.70%. Regarding marital status, 170 postdocs were married (46.32%), and 197 were single (53.68%). Considering the type of doctoral-granting institution, 341 individuals (92.92%) graduated from institutions in China, while 26 (7.08%) earned their doctoral degrees abroad. By academic discipline, 303 postdoctoral researchers (82.56%) belonged to the sciences, engineering, agriculture, and medicine fields, while 64 (17.44%) were in the humanities and social sciences.

### 3.2 Variable measurement and scale reliability tests

#### 3.2.1 Independent variable: WS

WS is a powerful guarantee to enhance postdoc CG, expressed explicitly in three sub-dimensions: mentor, institutional, and peer support. (1) Mentor support: Encompassing six items from the mentor support scale developed by Overall et al. (2011), this dimension measures the degree of assistance from mentors in areas such as expanding social networks, job recommendations and career development opportunities, academic exchange opportunities, project/grant applications, setting successful examples, and guidance on balancing work and life. (2) Institutional support: Assessed by three items derived from a questionnaire developed by Eisenberger et al. (1986), this dimension evaluates the organizations’ recognition and support of individual efforts and goals. (3) Peer support: Comprising three items selected from a six-item scale developed by Hobman et al. (2009), which assess the alignment of the attitudes and values of colleagues’ work with their own and the degree of care and concern shown by peers. Specifically, we utilized established scales, selecting high-factor loading items tailored to the Chinese postdoctoral working situation. Each item was tailored to suit the unique context of postdoctoral researchers in China. All items in the independent variables were measured using a 5-point Likert scale (1 = Strongly Disagree, 5 = Strongly Agree). A higher score indicates a more robust perception of WS. In this study, the Cronbach’s alpha coefficient for the WS scale was 0.934. The internal consistency coefficients for the mentor, institutional, and peer support subscales were 0.952, 0.930, and 0.868, respectively, indicating high-scale reliability.

#### 3.2.2 Mediating variables: PsyCap and WLB

In this investigation, PsyCap and WLB serve as mediators. To measure PsyCap, we employ the positive psychological capital questionnaire, developed by Zhang et al. (2010). The questionnaire includes four dimensions: optimism, resilience, self-efficacy, and hope. In our study, we selected four items with higher loadings, following the operational approach established by Zhou et al. (2018). These questions were scored on a 5-point Likert scale, with higher scores indicating higher positive tendencies in PsyCap. In previous studies, the scale demonstrated good reliability. In the present research, the scale showed a Cronbach’s alpha of 0.871, indicating good consistency among the items.

The WLB was derived from three high-factor loading items selected from the work-family balance scale developed by Grzywacz and Marks (2000). These criteria consider the impact of one’s professional pursuits on personal or family life, how personal or family life can affect professional pursuits, and the individual’s confidence in achieving professional success and personal fulfillment. Using a 5-point Likert scale, the responses showcased strong reliability with a Cronbach’s alpha of 0.879.

#### 3.2.3 Dependent variable: CG

The dependent variable is the CG experienced by postdoctoral researchers. This study measured CG using a simplified version of five items from Weng and McElroy’s (2012) 15-item scale. These selected items focus on evaluating key aspects: the development of professional skills, the availability of growth opportunities, the alignment with career goals, the sense of achievement derived from research milestones, and the belief in the significance of one’s research trajectory. We employed well-established scales, choosing items with high factor loadings relevant to the Chinese postdoctoral context. Each of these items was customized to reflect the distinctive experiences of postdoctoral researchers in China. Using a 5-point Likert scale, the responses showcased strong reliability with a Cronbach’s alpha of 0.914.

#### 3.2.4 Controlled variables

Consistent with the established literature, we integrated a set of control variables into our analytical model (Chen and Zhang, 2022; Zhao and Zhang, 2023). We considered the gender of the postdoctoral researcher (With females designated as 0 and males as 1), marital status (Using 0 for single and 1 for married individuals), type of doctoral-granting institution (Using 0 for institutions outside of China and 1 for institutions within China), and the disciplinary domain (Where 0 represents humanities and social sciences, while 1 denotes fields of science, technology, agriculture, and medicine).

### 3.3 Data analysis methods

We relied on SPSS 27.0 and Mplus 8.0 software packages for data analysis. Our preliminary examinations encompassed several diagnostic tests: reliability was assessed through Cronbach’s α coefficient; potential common method biases were identified; the data’s normal distribution was scrutinized; and we executed both Pearson correlation analysis and confirmatory factor analysis to gage the data’s suitability for further investigation. Using hierarchical regression, we examined the direct, mediating, and chain-mediating effects of postdoctoral PsyCap and WLB on the relationship between WS and CG. The Bootstrap method was subsequently employed to reaffirm the significance of mediation and chain mediation effects and elucidate any distinctions between the various established pathways.

## 4 Results

### 4.1 Assessment of common method bias

The data in this research are derived solely from self-reported accounts of the postdoctoral community. Although the data collection process was implemented in stages, it may still be susceptible to common method bias. For the study, Mplus 8.0 was used to conduct a Harman single-factor test on the 24 items to investigate potential bias. Following the approach of Zhou and Long (2004), we examined the data by setting the number of common factors at one. The fit indices of the single-factor model were found to be unsatisfactory (χ^2^/df = 9.973, CFI = 0.723, TLI = 0.677, RMSEA = 0.156), referencing the standards proposed by Harris and Mossholder (1996) and Long et al. (2002). Furthermore, to more accurately assess common method bias, this study employed the latent variable control method to establish a common method factor and subsequently formed a five-factor model (χ^2^/df = 7.188, CFI = 0.802, TLI = 0.777, RMSEA = 0.130). The results indicated that the fit indices of the five-factor model did not significantly outperform the four-factor model (χ^2^/df = 2.918, CFI = 0.942, TLI = 0.931, RMSEA = 0.072). This suggests that the data used in this study do not exhibit severe common-method bias, with the bias remaining within acceptable limits.

### 4.2 Confirmatory factor analysis

This study employed Mplus 8.0 to conduct confirmatory factor analysis (CFA) and evaluate discriminant validity among the measured variables. Based on a four-factor theoretical model (WS, PsyCap, WLB, and CG), we constructed the following competing models: a three-factor Model 1 (WS + PsyCap, WLB, CG), a three-factor Model 2 (WS + WLB, PsyCap, CG), a two-factor model (WS + PsyCap + WLB, CG), and a single-factor model (WS + PsyCap + WLB + CG). Comparing these models, the results favored the four-factor model as the most optimal (χ^2^ = 671.124, χ^2^/df = 2.918 < 3, CFI = 0.942 > 0.9, TLI = 0.931 > 0.9, RMSEA = 0.072 < 0.08). As shown in Table 1. This model outperformed the competing models, validating the discriminant validity among the core research variables of WS, PsyCap, WLB, and CG.

**Table 1:** Confirmatory factor analysis.

Model	χ^2^	df	χ^2^/df	CFI	TLI	RMSEA
Four-Factor	671.124	230	2.918	0.942	0.931	0.072
Three-Factor_1	1231.859	233	5.287	0.870	0.846	0.108
Three-Factor_2	1247.192	233	5.353	0.868	0.843	0.109
Two-Factor	1361.308	235	5.672	0.854	0.832	0.113
Single-Factor	2363.592	237	9.973	0.723	0.677	0.156

### 4.3 Analysis of variable correlation

The WS, PsyCap, WLB, and CG variables followed a normal distribution. The Pearson correlation analysis between each pair of variables is presented in Table 2. There was a significant positive correlation between WS and PsyCap (β = 0.606, *p* < 0.01), WLB (β = 0.643, *p* < 0.01), and CG (β = 0.488, *p* < 0.01). Similarly, PsyCap showed a significant positive correlation with both WLB (β = 0.507, *p* < 0.01) and CG (β = 0.488, *p* < 0.01). A notable positive correlation was also between WLB and CG (β = 0.442, *p* < 0.01). These results suggest that the relationships among the variables are broadly consistent with the hypothesized expectations, laying the foundation for subsequent chain mediation effect analyses.

**Table 2:** Analysis of variable correlation.

Variable	1	2	3	4
1. WS	1			
2. PsyCap	0.606**	1		
3. WLB	0.643**	0.507**	1	
4. CG	0.488**	0.488**	0.442**	1

### 4.4 Analysis of the pathways influencing CG

**Figure 2:**
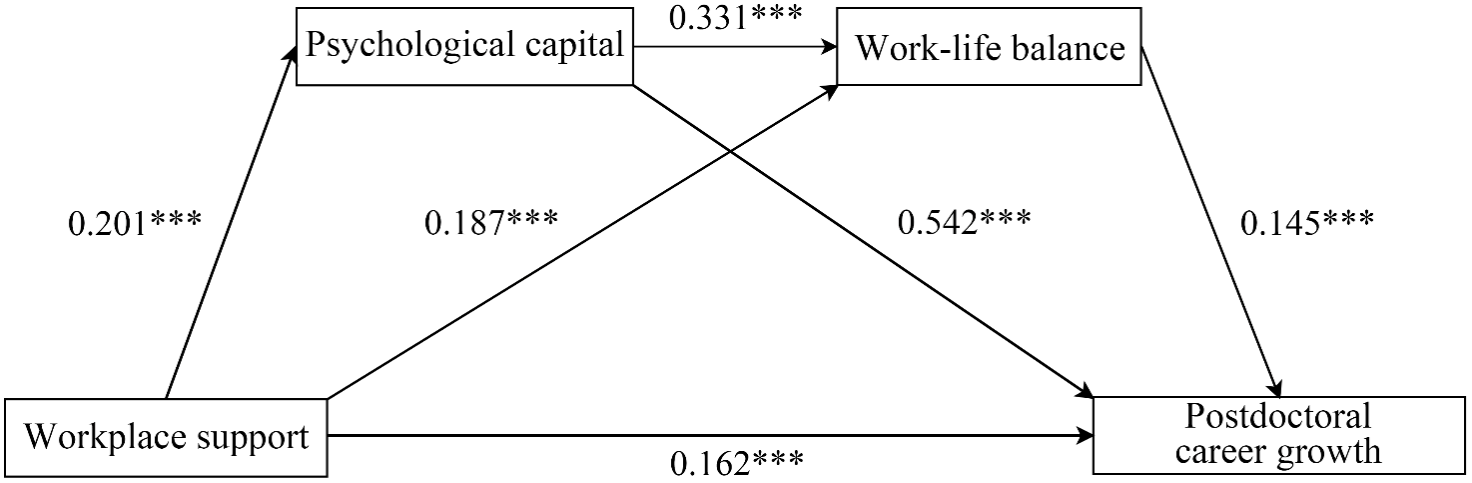
Role paths between variables.

In this study, hierarchical regression and Bootstrapping techniques using SPSS 27.0 were employed to investigate the relationship between WS, PsyCap, WLB and CG. As shown in Table 3. Firstly, the influence of WS on CG was examined. The results supported Hypothesis 1, showing a positive association between WS and CG (β = 0.162, SE = 0.023, *p* < 0.001). Next, the impact of WS on PsyCap and WLB was assessed. Consistent with Hypothesis 2a, WS positively influenced PsyCap (β = 0.201, SE = 0.018, *p* < 0.001). Similarly, in line with Hypothesis 3a, WS was found to have a positive effect on WLB (β = 0.187, SE = 0.027, *p* < 0.001). The relationship between PsyCap and both WLB and CG was then evaluated. The results provided evidence for Hypothesis 4a, indicating a significant positive relationship between PsyCap and WLB (β = 0.331, SE = 0.069, *p* < 0.001). Additionally, supporting Hypothesis 2b, PsyCap was significantly positively associated with CG (β = 0.542, SE = 0.056, *p* < 0.001). Finally, the analysis examined the influence of WLB on CG, validating Hypothesis 3b with a positive association (β = 0.145, SE = 0.041, *p* < 0.005). The results of the model path analysis are shown in Figure 2.

**Table 3:** Hierarchical regression analysis for direct and mediated effects tests.

Variable	CG	PsyCap	WLB	CG
	Model 1	Model 2	Model 3	Model 4
Gender	0.028	0.166^**^	0.060	−0.079
Marital status	0.041	0.114^*^	0.044	−0.032
Doctoral-granting institution	−0.028	−0.008	−0.018	−0.020
Discipline type	−0.046	−0.105	0.056	0.008
WS	0.308^***^	0.201^***^	0.187^***^	0.162^***^
PsyCap			0.331^***^	0.542^***^
WLB				0.145^**^
*R* ^2^	0.369	0.287	0.291	0.538
*F*	42.225^***^	29.000^***^	24.657^***^	59.654^***^

** indicates a significant correlation at the 0.01 level (two-tailed).

Furthermore, the mediation analyses for Hypotheses 2c and 3c and the chain-mediating effect proposed in Hypothesis 4b, were conducted; we utilized the PROCESS Model 6 for testing, with Bootstrap resampling set to 5,000 iterations. As detailed in Table 4, the results indicate that the total effect of WS on postdoctoral CG was 0.308, of which the direct effect was 0.162. Both PsyCap and WLB of postdoctoral researchers significantly played pivotal mediating roles between WS and CG, registering a combined mediating effect of 0.146, representing 47.403% of the total effect. After delving deeper into the mechanisms, we were able to identify three distinct mediating paths: WS influencing CG through PsyCap (β = 0.109), through WLB (β = 0.027), and a chained mediation path through PsyCap followed by WLB (β = 0.010). All three paths were statistically significant at the 95% confidence interval, supporting Hypotheses H2c, H3c, and H4b. The hypotheses proposed above are tested, as shown in Table 5. In terms of effect sizes, PsyCap served as a mediator for 35.390% of the total effect, while the path through WLB accounted for 8.766% and the combined chain mediation path represented 3.237%. These proportions indicate that PsyCap is the primary mediator of WS on CG, followed closely by WLB mediation and their combined chain mediation.

**Table 4:** Bootstrap chained mediator effect test results.

Effect	Paths	ꞵ	SE	95% confidence interval		Efficiency ratio
				Lower	Upper	
Total effect		0.308	0.021	0.266	0.349	1
Direct effect	WS-CG	0.162	0.023	0.118	0.206	52.594%
Mediation effect		0.146	0.020	0.107	0.184	47.403%
Ind1	WS-PsyCap-CG	0.109	0.017	0.077	0.144	35.390%
Ind2	WS-WLB-CG	0.027	0.009	0.009	0.046	8.766%
Ind3	WS-PsyCap-WLB-CG	0.010	0.004	0.003	0.020	3.237%

**Table 5:** Summary of results.

Hypotheses	Findings
H1: WS is positively associated with CG of postdoc	Supported
H2a: WS is positively associated with PsyCap of postdoc	Supported
H2b: PsyCap is positively associated with CG of postdoc	Supported
H2c: PsyCap mediates the impact of WS on CG	Supported
H3a: WS is positively associated with WLB of postdoc	Supported
H3b: WLB is positively associated with CG of postdoc	Supported
H3c: WLB mediates the influence of WS on CG	Supported
H4a: PsyCap is positively associated with WLB of postdoc	Supported
H4b: WS influences CG through a chain-mediating effect of PsyCap and WLB	Supported

## 5 Discussion

### 5.1 Theoretical implications

The present study proposes a chained mediation model based on survey data from a specific population, from a COR theory perspective, investigating the impact and mechanisms of WS on postdoctoral PsyCap, WLB, and CG. Empirical evidence suggests that promoting a positive psychological state, comprising PsyCap and WLB, through multidimensional organizational support has a positive impact on the academic performance of young scholars. It enriches the theoretical examination of the application and development of COR theory in individual career growth. It provides a deeper understanding of the pathways facilitating CG, offering critical insights into promoting quality postdoctoral occupational health development, and organizational support climate creation.

#### 5.1.1 The direct positive influence of WS on CG

Empirical analysis has demonstrated that WS is directly and positively associated with CG among postdoctoral researchers, indicating that such support is a practical external resource in the framework of COR theory. It helps counter pressures arising from individual resource deficiencies, enhancing postdoctoral CG throughout their tenure. Historical research corroborates these findings. For example, De Janasz and Sullivan (2004) discussed the role of multiple mentoring within the academic realm, emphasizing the importance of nurturing supportive mentor-mentee relationships for developing early-career academics. Furthermore, a study on job satisfaction revealed that positive feedback from colleagues and organizations could spur increased creativity among less satisfied employees, thus catalyzing their career growth (Zhou and George, 2001). To summarize, existing studies on postdoc or PhD students have similarly identified WS as a crucial catalyst for their CG (Louis et al., 2007; Gloria and Steinhardt, 2017; Hafsteinsdóttir et al., 2017).

#### 5.1.2 The mediating role of PsyCap and WLB in CG

Path analysis indicates that WS influences CG, notably through its effects on PsyCap and WLB. Specifically, the mediating effect of PsyCap accounts for 35.39% of the total effect, which signifies its pivotal role as an intermediary in the relationship between WS and CG. On the one hand, WS provides an environment that meets the fundamental psychological needs of postdocs. As this support increases, an individual’s autonomy, competence, and relatedness needs are met, strengthening PsyCap (Deci and Ryan, 1985). This solidifies the position of WS as a crucial determinant in the enhancement of PsyCap (Stenling et al., 2021). On the other hand, the study confirms previous research indicating that developing an individual’s Psycap improves their CG (Saleem et al., 2022c). Having high levels of PsyCap lays the foundation for cultivating positive research behavior and attitudes (Scheier and Carver, 1985), acting as a buffer against negative stressors (Masten and Reed, 2002), helping postdocs navigate academic challenges, surmount research obstacles, and steadily advance their careers (Seligman et al., 2005). Boosting postdoctoral PsyCap is an effective strategy to enhance their CG amidst challenges in postdoctoral academic professional socialization.

Further path analysis revealed that WLB is an additional mediating factor, which accounts for 18.49% of the total effect. The literature emphasizes the critical role of WLB in the relationship between WS and CG (Clark, 2000; Greenhaus et al., 2003). Firstly, WS is positively predictive of WLB, a result that is consistent with prior research (Abendroth and Den Dulk, 2011; Amazue and Onyishi, 2016; Sheikh, 2022). Support from mentors, institutions, and peers helps postdocs establish and maintain work-life boundaries, thereby attenuating work-life conflicts (Clark, 2000). Academic network and expert guidance of mentors ensure the efficient completion of research tasks, mitigate excessive overtime, and alleviate concerns related to career stability (Ghosh and Reio, 2013). Institutional recognition of postdoctoral academic contributions enhances their job satisfaction and career identification (Weng and Zhu, 2020). Peer cooperation and resource sharing can minimize task redundancy and conflict, while a harmonious, collaborative team environment ensures smooth work progression, staving off unnecessary interpersonal disputes and stress (Weaver et al., 2021). Postdocs in supportive environments are more likely to perceive a balance between scientific work and personal life. Secondly, WLB has largely been ignored as a potential approach to providing postdocs with a balanced and suitable environment for growth (Au and Ahmed, 2015; Russo et al., 2016). Our study emphasizes the critical transduction role of WLB between WS and CG. The work-life conflict has been linked to lower job satisfaction, academic burnout, and decreased self-efficacy (Fox et al., 2011). Consequently, WLB is a constructive buffer against occupational pressure (Bozeman and Gaughan, 2011). It can act as an initial resource to deter resource loss and an acquired resource to catalyze more significant resource gains, hence driving CG (Ten Brummelhuis and Bakker, 2012).

#### 5.1.3 The chain-mediation effect of PsyCap and WLB in CG

The model test results illustrate that WS impacts postdoctoral CG through the chain-mediation effect of PsyCap and WLB. These analyses support social support theory, indicating that WS protects postdoctoral well-being, enhances PsyCap and WLB (Lakey and Orehek, 2011). Research on PsyCap has neglected the work-related social domain of the work-family interface. This construct extends to the postdoctoral social domain, including a positive appraisal of work-life interaction. The current study fills the knowledge gap between PsyCap, WLB, and CG research. Therefore, the pathway of “PsyCap-WLB” is essential in enhancing the CG of postdoctoral researchers. Such conclusions align with the gain spiral effect mentioned in COR theory. When robust external provisions timely replenish the individual resources expended during postdoctoral socialization to academic profession, it triggers a chain reaction of positive emotions and quickly replenishes resources, which in turn has a positive impact on CG (Hobfoll, 2001; Hobfoll et al., 2018).

### 5.2 Practical implications

Postdoc is designed to accelerate the evolution of doctoral graduates into early career researchers, facilitating their progression from doctoral students to professional researchers. This period signifies a transition from a passive to an active research approach and a shift from uncontrolled academic growth to intentional academic development. During the postdoctoral phase, individuals typically experience a significant improvement in their ability to conduct independent research effectively, including topic selection, competitive research endeavors, research organization, and the high-quality completion of research topics. As such, the postdoctoral phase is characterized by pressing time constraints and challenges related to survival prospects, academic growth, and the struggle for autonomy and independence in the professional academic milieu (Weidman and Stein, 2003).

Drawing from social support theory, protective factors associated with positive outcomes are believed to buffer, moderate, or counteract the adverse effects of stress, setbacks, or adversities, promoting better individual adaptation (Rutter, 2000). In the postdoctoral context, elements such as a supportive mentoring team, dedicated postdoctoral workstations, and a robust network act as protective factors for psychological well-being. These institutions can take specific actions, including developing structured mentorship programs, establishing dedicated workspaces for postdoctoral researchers, and fostering a collaborative and supportive culture among colleagues. Individuals perceiving such support are better equipped to manage stress and minimize adaptive challenges, subjectively promoting CG (Lakey and Orehek, 2011). Additionally, the Gain Paradox principle suggests that the detrimental impacts of research-related stresses can amplify the positive effects of WS (Hobfoll, 2001). A powerful perception of this support deepens the emotional attachment of postdoctoral researchers to their stations, reinforcing their academic professional identity. This, in turn, generates intrinsic solid motivation, dissipating external threats to their occupational mental health. The socialization process has been conclusively determined to be a critical factor in doctoral success (Turner and Thompson, 1993). Thus, timely and potent WS emerges as an essential tool for research institutions, aiding postdocs in tackling the challenges of academic professional socialization and promoting their careers in academia, paving the way for a successful academic career by shaping their academic aspirations and identity.

A robust support system, including mentors, institutions, and peers, can make all the difference in a postdoctoral research journey. With this support, postdoc can reach their full potential and accomplish remarkable feats in their academic career. When transposed to broader academic and professional contexts, the principles remain salient. While central to the academic landscape as guidance, mentorship can be seen in business and industry as an expression of developmental human resource management practices (Kuvaas, 2007). By creating an environment where experienced individuals mentor novices, institutions can enhance professional development and knowledge transfer. Furthermore, IS, spotlighted in our research, suggests that all types of institutions, whether they are academic hubs, emerging startups, or large corporations, can greatly benefit from creating strong support systems, whether through training programs, allocation of resources, or development of policies. Lastly, the resonance of PS speaks to the universal truth: a collaborative environment infused with mutual respect and camaraderie is instrumental in advancing both individual and collective growth.

## 6 Limitations and future research directions

Our study has several limitations, which may lead to implications for future research. First, distributing questionnaires predominantly via personal emails and social networking platforms posed challenges in collection, leading to a relatively limited sample size, which can affect the generalizability of the study conclusions. Adopting a longitudinal design with time-lagged data can help future studies better understand potential causal relationships. Second, while PsyCap and WLB accounted for 47.403% of the total effect of WS on postdoctoral CG, a significant direct effect remains, suggesting the potential existence of other mediating factors. Individual behavioral variables closely related to work situations should be explored in future research. Moreover, the reliance on self-assessment questionnaires completed by postdocs could introduce biases due to the specific circumstances or mindsets of the respondents at the time of completion. Future studies may benefit from incorporating third-party evaluations in subsequent research to enhance the findings’ reliability.

## Data availability statement

The raw data supporting the conclusions of this article will be made available by the authors, without undue reservation.

## Ethics statement

The studies involving humans were approved by the Ethics Committee at the Beijing Forestry University. The studies were conducted in accordance with the local legislation and institutional requirements. Written informed consent for participation was not required from the participants or the participants’ legal guardians/next of kin in accordance with the national legislation and institutional requirements.

## Author contributions

XL: Conceptualization, Data curation, Formal analysis, Methodology, Software, Validation, Writing – original draft. AH: Conceptualization, Data curation, Formal analysis, Methodology, Validation, Visualization, Writing – original draft. HS: Conceptualization, Funding acquisition, Investigation, Project administration, Resources, Supervision, Writing – review & editing. ZW: Resources, Supervision, Writing – review & editing.
